# Healthcare simulation and contextually relevant primary care training curricula in Africa

**DOI:** 10.4102/safp.v68i1.6281

**Published:** 2026-05-14

**Authors:** Marvin J. Jansen, Tasleem Ras

**Affiliations:** 1 Department of Health Sciences Education, Faculty of Health Sciences, University of Cape Town, Cape Town, South Africa; 2 Division of Family Medicine, Department of Family, Community and Emergency Care, Faculty of Health Sciences, University of Cape Town, Cape Town, South Africa

**Keywords:** Healthcare simulation, Primary Health Care, contextually relevant curricula, curriculum development, Family medicine

## Abstract

Healthcare simulation has emerged as a powerful educational strategy for primary care clinician training across Africa. Simulation-based learning, ranging from high-fidelity manikins to standardised patient scenarios; enables safe, hands-on practice of clinical skills tailored to local health priorities. In undergraduate, postgraduate, and in-service settings, simulation is being used to strengthen competencies in managing common and emergency conditions without risk to patients. This article explores how simulation pedagogy contributes to contextually relevant curricula for Primary Health Care (PHC), highlighting examples of successful programmes in African countries. We discuss the evidence of improved skills, teamwork, and patient outcomes from simulation-based training, alongside the challenges of cost, faculty development, and resource constraints. Innovative solutions such as low-cost simulators, in situ training, and international partnerships are reviewed.

**Contribution:**

With growing support from frameworks such as the World Health Organization and national initiatives, simulation offers adaptable, feasible, and pedagogically rich approaches to train competent primary care providers in diverse African contexts.

## Introduction

Primary Health Care (PHC) is the backbone of health systems in Africa, yet training the clinicians who deliver PHC faces unique challenges.^1^ Many African medical and nursing programmes must prepare graduates for broad generalist roles in communities with limited resources. Traditional curricula have often been theory-heavy and hospital-centric, which can leave new clinicians underprepared for the realities of front-line primary care.^2,3^ Healthcare simulation offers a pedagogical approach well-suited to bridge this gap. Simulation creates a safe, controlled learning environment where trainees can practice clinical and decision-making skills on life-like patient scenarios, receive feedback, and build confidence without risking patient harm.^4^ Notably, simulation-based education (SBE) has been identified as a priority by the World Health Organization (WHO) for improving health workforce training in low- and middle-income countries.^5^ A recent global consensus also emphasises that simulation plays a pivotal role in addressing healthcare challenges, reducing training inequities, and ultimately improving patient outcomes.^6^

Across Africa, interest in simulation as a teaching method is growing among faculty and learners alike.^4,7^ Simulation allows educators to design curriculum content that reflects local epidemiology, common conditions, and resource constraints. For example, scenarios can be tailored to manage illnesses like obstetric emergencies, paediatric febrile convulsions, or tuberculosis (TB); aligning training with the health issues clinicians will face in their communities. By reproducing realistic primary care settings (from rural clinics to urban health centres) within a simulation, trainees learn to navigate the challenges of their working environment, including limited equipment or the need for improvisation. This contextualised learning is critical for relevance, instead; of relying on imported case studies or hospital-based algorithms, simulation curricula in Africa can integrate community health priorities, cultural considerations, and locally available treatments.^8^ Such alignment with context helps to ensure that graduates and health workers are practice-ready for the African PHC context upon completion of training.^9^ A scoping review found that a major challenge in developing countries has been the *lack of a contextual curriculum*, and that simulation provides an opportunity to redesign training in line with actual local needs.^4^ By focusing on realistic cases and environments, simulation-based training builds competencies that directly translate to improved primary care service delivery.

## Simulation in undergraduate education

Higher health educational institutions schools in Africa are increasingly adopting simulation to enhance undergraduate training in primary care competencies. Medical and nursing students often have limited clinical exposure or supervision in busy health facilities.^10^ Simulation helps to fill this gap by allowing deliberate practice of clinical skills before encountering real patients. Universities have set up skills labs and simulation centres where students can learn procedures and participate in scenario-based drills for primary care situations. This educational approach links theory to practice, applying clinical guidelines in a life-like scenario.^11,12,13,14^

In African undergraduate programmes, simulation’s pedagogical strengths include the ability to standardise learning experiences (every student can manage the same scenario of, say, an adult with human immunodeficiency virus and/or TB co-infection), the opportunity for immediate feedback during debriefings, and the fostering of clinical reasoning and teamwork early in training. Students can repeatedly practice high-stakes, low-frequency events that are crucial in primary care, such as neonatal resuscitation or anaphylaxis management, so that when these events occur in real life, the new clinician is better prepared.^15,16,17^ Evidence suggests that simulation-based training improves knowledge, clinical performance, and learner confidence.^14^ It also reduces the burden on real patients as teaching subjects. By the time students graduate, exposure to robust simulation curricula may provide opportunities to develop practical skills and decision-making in contextually relevant scenarios. This may facilitate the transition into internship or community service and support preparedness for delivering care at the primary level.

## Simulation in postgraduate and in-service training

Beyond pre-service education, simulation has proven highly valuable in postgraduate training (such as family medicine residencies) and in-service professional development for primary care teams. Clinicians who are already practising in clinics and district hospitals often face emergencies and complex cases with limited specialist support. However, opportunities for further hands-on training can be scarce, and many practitioners feel ill-equipped for certain acute care situations in primary care.^18^ Simulation-based drills and courses offer a way to continuously upskill these providers in a practical, engaging manner.

In South Africa, family physicians in the Tshwane District recognised gaps in the emergency skills of clinicians at primary care facilities.^14^ Routine Patient Safety Incident audits had revealed adverse outcomes in clinics because of delayed or suboptimal emergency care. To tackle this, two family physicians initiated a programme of monthly on-site emergency simulation drills at community health centres. Over 18 months (2022–2023), they conducted 12 simulation training sessions across various clinics, each session focusing on a common emergency scenario drawn from real cases seen in primary care.^18^ These simulations were multidisciplinary, involving nurses, doctors, and other staff working as a team to manage the simulated patient, followed by a debriefing discussion. Importantly, the cases were chosen based on local incident patterns, making the training highly relevant to the participants’ daily work.

The outcomes of this intervention were notable. Staff reported improved clinical skills and greater confidence in handling emergencies after the drills. Teamwork was enhanced, participants learned to communicate and coordinate better as a unit when responding to acute emergencies or critical illnesses. The drills also served as a catalyst for reviewing the clinics’ preparedness: during debriefings, teams identified missing emergency equipment or drugs and took steps to stock or maintain them. In essence, these simulation exercises not only taught clinical management but also prompted system improvements. This Tshwane programme exemplifies how in-service simulation can address context-specific training needs and empowers primary care teams to respond effectively.^18^

Maternal and child health is another domain where simulation-based in-service training has thrived across Africa. A prominent example is the Helping Babies Breathe (HBB) programme; a short simulation-based course in neonatal resuscitation for birth attendants. Helping Babies Breathe uses low-cost infant manikins and role-play to teach the initial steps of newborn care and resuscitation in resource-limited delivery settings. It has been implemented widely in African countries (including Tanzania, Kenya, Uganda, Ghana and others) to improve birth outcomes. Studies have shown significant impact: an analysis of ~80 000 births in Tanzania after HBB training demonstrated a 47% reduction in early neonatal deaths within 24 h, as well as a 24% reduction in fresh stillbirth rates.^16,19,20,21,22,23^ These remarkable outcomes, achieved with a 1-day course and simple bag-and-mask equipment highlight how simulation training targeted to a local priority (newborn asphyxia) can translate into lives saved. Collectively, these maternal-newborn simulation programmes show how standardised, evidence-based curricula can be adapted for low-resource contexts (using affordable simulators and focusing on basic life-saving skills) and delivered at scale to in-service clinicians, with measurable improvements in health outcomes.

Family medicine postgraduate training programmes are also leveraging simulation. In South Africa, specialist family physician trainees must be equipped for both clinical and leadership roles in the district health system. Simulation is used in some training sites to practice complex primary care scenarios, including communication challenges (breaking bad news, counselling families) using standardised patient actors, and clinical procedures relevant to family medicine.^18^ For example, registrars might participate in simulated clinic days where they encounter a series of patients presenting with undifferentiated problems common in primary care. These simulations build skills in diagnosis, holistic management, and teamwork with nurses. They also allow assessment of the registrars in a standardised way.^24^ The integration of simulation into formal postgraduate curricula is still in early stages in many African countries, often limited by resource and faculty constraints, but momentum is growing.

From rural health posts to urban clinics, simulation-based training has been introduced at all levels of the primary care workforce development pipeline. These examples illustrate the diversity of approaches, from brief on-site drills to large-scale national programmes, and their outcomes in strengthening primary care delivery.

## Challenges and innovations in implementation

Implementing SBE in African contexts comes not only with several challenges but also spurs innovative solutions. One of the foremost challenges are resource limitations; appropriate equipment and dedicated simulation facilities are expensive. Many institutions simply cannot afford these advanced technologies. Simulation in Africa has unique hurdles related to the *exorbitant cost* of modern manikins and audiovisual equipment.^13,18^ To address this, educators have turned to low-cost and low-tech simulation tools. Programmes such as HBB explicitly use affordable models (neonatal and obstetric simulators that cost a fraction of high-end manikins) and even simple props to mimic clinical scenarios. In settings without any manikins, trainers improvise, for example, using a balloon or cloth to simulate an organ, chicken bones to simulate human bones, or a volunteer acting as a patient.

A closely related challenge is the dearth of trained simulation educators. Effective simulation-based training requires facilitators skilled in scenario design, operation of simulators, and debriefing techniques. Many African health training institutions lack personnel with formal training in these areas.^4^ Performance anxiety among both trainers and trainees when using new simulation methods can also impede uptake.^25^ To overcome this, capacity-building initiatives have been key. The ‘Simulation Launchpad’ course, for instance, was developed to build a cadre of simulation instructors in Africa, graduating dozens of educators from nine countries in just 2 years.^13^ By providing workshops on simulation methodology and mentoring educators through running their first scenarios, such programmes help to create local champions who can spread simulation at their home institutions. The virtual faculty development partnership in Kenya is another model, where distance learning and mentorship enabled faculty to gain simulation skills without costly travel.^26^

Another significant challenge is curriculum integration and curricular overload. Many health training programmes already have dense curricula, and it can be difficult to allocate time for simulation sessions or drills. In some cases, educators may be unsure how to fit simulation into exam-driven programmes. However, there is growing acknowledgement that simulation should not be seen as an ‘add-on’ but rather as an integral pedagogy to achieve competency-based outcomes.^27^ Academic leadership in Africa is increasingly showing interest and urgency in adopting simulation in curricula and allocating funds for it.^4^ For example, several South African medical schools have embedded simulation in their curricula for clinical skills learning and Objective Structured Clinical Examination (preparation). One strategy has been to replace some passive learning activities (such as lectures) with simulation-based exercises that teach the same content more actively. The trend towards competency-based education and workplace-based assessment in family medicine and other disciplines also aligns with simulation; tasks that trainees must be entrusted to perform (entrustable professional activities) can be practised and evaluated in simulation before real-world execution. By clearly demonstrating the link to improved patient safety and clinical competence, advocates for simulation have made a case that dedicating time to simulation is worthwhile. In fact, in developing countries, reframing simulation not only as an educational tool but as a *patient safety intervention* can help garner administrative support,^4^ since better-trained clinicians will likely commit fewer errors.

Logistical and systemic challenges also exist. These include scheduling difficulties (e.g. shift workers finding it hard to attend training), high staff turnover (so skills gained may be lost if trained staff leave), and geographic dispersion (making it hard to bring learners to a central simulation centre). Innovative solutions have emerged in response. Many programmes opt for in situ simulation; conducting training on-site in the clinic or ward where staff work, to avoid the logistical hurdles of travel and scheduling. A scoping review of emergency obstetric simulation in sub-Saharan Africa found that on-site training can reach more staff and generate more relevant organisational improvements compared to off-site simulation in a distant centre.^14^ In situ sessions are often shorter ‘drills’ that can be performed during shifts, thus minimising disruption. To address retention and staff turnover, the concept of low-dose, high-frequency training (short, repeated simulations) has been employed, as well as a ‘train-the-trainer’ approach so that each facility has someone who can continue running simulations for new staff.

Cultural acceptance is an often under-discussed challenge; some learners initially find simulation odd or fear that poor performance in a simulation might be punitive. Building a culture of safety in simulation, emphasising confidentiality, providing a safe learning environment with a focus on learning, is essential.^25^

The emergence of artificial intelligence (AI) presents new opportunities and challenges for SBE in Africa. Artificial intelligence-enabled tools, such as virtual patients and automated feedback systems, have the potential to enhance scalability, provide personalised learning, and extend access to training in resource-constrained settings. These innovations may complement traditional simulation approaches by enabling continuous, self-directed practice. However, their implementation must be approached cautiously, considering constraints related to digital infrastructure, data governance, cost, and equity. There is also a need to ensure that AI-driven solutions remain contextually relevant and do not replace the critical role of human facilitation, particularly in debriefing and reflective learning. As such, AI should be viewed as an emerging adjunct to simulation, with further research needed to understand its effectiveness and applicability within African primary care contexts.

Finally, language and context barriers are addressed by localising scenarios. In multilingual settings, simulations can be run in the local language to mimic real patient interactions. Scenarios also incorporate sociocultural elements, for instance, a simulation of a community health worker visit might include managing a family’s traditional beliefs along with clinical care, or a chronic disease management scenario may involve addressing patients’ use of herbal traditional remedies. These nuances make the curriculum more relevant and culturally sensitive, something traditional textbooks cannot easily provide.

While Africa’s adoption of simulation in primary care training faces obstacles such as cost, limited expertise, and logistical issues, these challenges are being met with innovations; low-cost simulation models, faculty development and networking, on-site and modular training designs, curriculum reforms, and creative improvisation. The trajectory is clearly towards growth in simulation use, driven by recognised gains in training quality. Overcoming implementation hurdles means that more African clinicians can say their first intubation, first management of eclampsia, or first paediatric resuscitation was carried out in simulation; ensuring they are better prepared when the real moment comes.

## Policy frameworks and regional initiatives

The expansion of SBE in Africa has been supported by several frameworks and initiatives at global, regional, and national levels. The WHO’s focus on transformative health workforce training has implicitly endorsed approaches such as simulation that improve competency and patient safety.^5^ This aligns with broader WHO frameworks on health systems, for example, the WHO Patient Safety curriculum^28^ encourages simulation for teaching safe practices, and WHO’s emergency care training packages incorporate scenario-based drills for frontline providers.^29^

Regionally, networks and professional bodies are emerging to champion simulation in African health education. The African Simulation Network and Morocco SIM are examples of such communities. Through social media and workshops, these networks have connected innovators from different countries, accelerating learning and collaboration.

In essence, the growth of simulation in Africa is not happening in isolation but is reinforced by a conducive policy environment and a spirit of collaboration. From the WHO’s high-level endorsement to grassroots educator networks, the pieces are in place for simulation to flourish as a standard part of primary care training. The challenge moving forward will be to maintain momentum, share successes and failures openly, and ensure that initiatives are scaled in a way that is locally led and contextually appropriate.

## Practical implications

Primary Health Care providers seeking to introduce or strengthen simulation in primary care can begin with simple, low-cost approaches. Starting with in situ simulations in existing clinical spaces, using common high-risk scenarios identified from routine practice or patient safety incidents, allows teams to train within their real context. Engaging multidisciplinary staff and incorporating structured debriefing after each session are key to maximising learning. Regular, short simulation drills can be more feasible than large-scale programmes and support ongoing skill development. Building local faculty capacity and linking with regional networks can further enhance sustainability and impact.

## Conclusion

In conclusion, healthcare simulation offers a feasible, adaptable, and effective approach to strengthen primary care training in Africa. It aligns with the continent’s push for high-quality, practical education that produces healthcare providers who are ready to serve communities from day one. As more success stories emerge, and as research continues to document outcomes and refine techniques, simulation is poised to become an indispensable element of curricula for medical, nursing, and other health professions in Africa. By embedding locally relevant simulation exercises in training, Africa’s educational institutions are not only teaching skills but also cultivating teamwork, communication, and critical decision-making under pressure, all of which are hallmarks of strong PHC. Ultimately, the expansion of simulation-based training contributes to building a confident and competent primary care workforce, which is essential for achieving better health outcomes across the continent. The challenge ahead will be to scale these efforts equitably and sustainably, ensuring that even the most resource-limited settings can benefit from this transformative educational methodology. With continued innovation, investment, and collaboration, simulation may play a central role in shaping the future of primary care clinician education in Africa.

## References

[1] World Health Organization (WHO). Operational framework for primary health care transforming vision into action. Geneva: WHO; 2018.

[2] Frenk J, Chen L, Bhutta ZA, et al. Health professionals for a new century: Transforming education to strengthen health systems in an interdependent world. Lancet. 2010;376(9756):1923–1958. 10.1016/S0140-6736(10)61854-5 21112623

[3] Reddi S, Javidi D. A critical narrative review of medical school curricula: Teaching methods, assessment strategies, and technological integration. Cureus. 2025;17(4):e82015. 10.7759/cureus.82015 40351904 PMC12065960

[4] Ismail FW, Ajani K, Baqir SM, Nadeem A, Qureshi R, Petrucka P. Challenges and opportunities in the uptake of simulation in healthcare education in the developing world: A scoping review. MedEdPublish. 2024;14:38. 10.12688/mep.20271.1 39257565 PMC11384200

[5] Robinson SJA, Ritchie AMA, Pacilli M, Nestel D, McLeod E, Nataraja RM. Simulation-based education of health workers in low- and middle-income countries: A systematic review. Glob Health Sci Pract. 2024;12(6):e2400187. 10.9745/GHSP-D-24-00187 39510603 PMC11666082

[6] Diaz-Navarro C, Armstrong R, Charnetski M, et al. Global consensus statement on simulation-based practice in healthcare. Adv Simul. 2024;9(1):19. 10.1186/s41077-024-00288-1 PMC11106913 38769577

[7] Swart R, Duys R, Hauser ND. Sass: South African simulation survey – A review of simulation-based education. South Afr J Anaesth Analg. 2019;25(4):12–20. 10.36303/SAJAA.2019.25.4.2191

[8] Schrewe B, Ellaway RH, Watling C, Bates J. The contextual curriculum: Learning in the matrix, learning from the matrix. Acad Med. 2018;93(11):1645–1651. 10.1097/ACM.0000000000002345 29979208

[9] Ray SC. Editorial: Innovative educational methods for Family Medicine and Primary Care training. Afr J Prim Health Care Fam Med. 2024;16(1): 4833. 10.4102/PHCFM.v16i1.4833 39846115 PMC11850998

[10] World Health Organization (WHO). Transforming and scaling up health professionals’ education and training in Africa. Brazzaville: WHO; 2013. 26042324

[11] Jansen MJ, Hartman N, Grant D. Undergraduate acute care clinical competencies for managing acute care cases in adult patients within a South African in-hospital environment: A modified Delphi Study. Afr J Emerg Med. 2024;14(3):231–236. 10.1016/j.afjem.2024.08.004 PMC11400605 39281663

[12] Livingston P, Bailey J, Ntakiyiruta G, Mukwesi C, Whynot S, Brindley P. Development of a simulation and skills centre in East Africa: A Rwandan-Canadian partnership. Pan Afr Med J. 2014;17:315. 10.11604/pamj.2014.17.315.4211 25328611 PMC4198314

[13] Park-Ross JF, Duys R, Espen B, Gray R, Jansen M. The Simulation Launchpad course: Building simulation capacity in Africa. Int J Healthc Simul. 2024:1–3. 10.54531/hfoy7377

[14] Van Tetering AAC, Ntuyo P, Martens RPJ, et al. Simulation-based training in emergency obstetric care in sub-Saharan and Central Africa: A scoping review. Ann Glob Health. 2023;89(1):62. 10.5334/aogh.3891 37780839 PMC10540704

[15] Mcgaghie WC, Issenberg SB, Barsuk JH, Wayne DB. A critical review of simulation-based mastery learning with translational outcomes. Med Educ. 2014;48(4):375–385. 10.1111/medu.12391 24606621

[16] Ersdal HL, Vossius C, Bayo E, et al. A one-day ‘Helping Babies Breathe’ course improves simulated performance but not clinical management of neonates. Resuscitation. 2013;84(10):1422–1427. 10.1016/j.resuscitation.2013.04.005 23612024

[17] Ersdal H, Mdoe P, Mduma E, et al. ‘Safer births bundle of care’ implementation and perinatal impact at 30 hospitals in Tanzania – Halfway evaluation. Children. 2023;10(2):255. 10.3390/children10020255 36832384 PMC9955319

[18] Eales O, Kruger A. Development of practical emergency simulation training in primary health care: Lessons learnt. Afr J Prim Health Care Fam Med. 2024;16(1):e1–e3. 10.4102/phcfm.v16i1.4404 PMC11151345 38832378

[19] Kamath-Rayne BD, Berkelhamer SK, Kc A, Ersdal HL, Niermeyer S. Neonatal resuscitation in global health settings: An examination of the past to prepare for the future. Pediatr Res. 2017;82(2):194–200. 10.1038/pr.2017.48 28419084

[20] Arlington L, Kairuki AK, Isangula KG, et al. Implementation of ‘Helping Babies Breathe’: A 3-year experience in Tanzania. Pediatrics. 2017;139(5):e20162132. 10.1542/peds.2016-2132 28557724

[21] Patel AB, Bang A, Kurhe K, Bhargav S, Hibberd PL. What Helping Babies Breathe knowledge and skills are formidable for healthcare workers? Front Pediatr. 2023;10:891266. 10.3389/fped.2022.891266 36793503 PMC9922883

[22] Msemo G, Massawe A, Mmbando D, et al. Newborn mortality and fresh stillbirth rates in Tanzania after helping babies breathe training. Pediatrics. 2013;131(2):e353–e360. 10.1542/peds.2012-1795 23339223

[23] Mayer M, Xhinti N, Dyavuza V, Bobotyana L, Perlman J, Velaphi S. Assessing implementation of Helping Babies Breathe program through observing immediate care of neonates at time of delivery. Front Pediatr. 2022;10:864431. 10.3389/fped.2022.864431 35547538 PMC9083269

[24] McGaghie WC, Issenberg SB, Petrusa ER, Scalese RJ. A critical review of simulation based medical education research 2003-2009. Med Educ. 2010;44(1):50–63. 10.1111/j.1365-2923.2009.03547.x 20078756

[25] Rudolph JW, Raemer DB, Simon R. Establishing a safe container for learning in simulation: The role of the presimulation briefing. Simul Healthc. 2014;9(6):339–349. 10.1097/SIH.0000000000000047 25188485

[26] Fant C, Olwala M, Laanoi GM, et al. Virtual faculty development in simulation in Sub-Saharan Africa: A pilot training for pediatricians in Kisumu, Kenya. Front Pediatr. 2022;10:957386. 10.3389/fped.2022.957386 36210954 PMC9538528

[27] Jansen M, Hartman N, Grant D. The role of medical simulation curriculum in developing acute care clinical competence in undergraduate medical students in South Africa [PhD thesis]. Cape Town: University of Cape Town; 2022. Available from: https://open.uct.ac.za/items/a15f73e3-63fb-4a61-bccd-b11fe51a6095

[28] World Health Organization (WHO). Patient safety curriculum guide: Multi-professional Edition. Geneva: WHO; 2011, p. 270.

[29] Ahun MN, Aboud F, Wamboldt C, Yousafzai AK. Implementation of UNICEF and WHO’s care for child development package: Lessons from a global review and key informant interviews. Front Public Health. 2023;11:1140843. 10.3389/fpubh.2023.1140843 36875409 PMC9978394

